# Assessing System Justification in Czech Population Using the System Justification Scale

**DOI:** 10.3390/ejihpe13090131

**Published:** 2023-09-13

**Authors:** Jiri Remr

**Affiliations:** INESAN (Institute for Evaluations and Social Analyses), Sokolovská 351/25, 18600 Prague, Czech Republic; jiri.remr@inesan.eu

**Keywords:** system justification, psychometrics, CFA, validity, rule compliance, social inequalities, justice, equity

## Abstract

System justification (SJ) is an important construct in social psychology that has received considerable attention over the past three decades. At the empirical level, system justification is examined by means of a specially developed System Justification Scale (SJS), which is designed to explain how individuals accept justice, whether they consider a given social order to be fair, how they evaluate the conditions in the country in which they live, how they accept social change, or to what extent they express compliance with established rules. System justification involves not only those who benefit from the existing social order, but also those who are disadvantaged. In their case, system justification mitigates negative perceptions of objective inequalities and asymmetries. Empirical evidence suggests that system justification may also be associated with higher perceived quality of life. The present study translated and validated the SJS, providing complex and detailed information on the psychometric properties of the scale. In addition, the scale’s internal consistency, unidimensionality, and construct validity were examined. The conclusions presented are based on the results of exploratory factor analysis, internal consistency assessment, analysis of variance, correlation analysis, and confirmatory factor analysis. Results were obtained from face-to-face survey data collected from a sample of 1419 individuals representing the Czech population aged 18–79 years. Since the SJS showed high internal consistency, adequately discriminated levels of system justification, and had robust psychometric properties, it could be recommended for further use.

## 1. Introduction

System justification theory has received considerable attention from researchers in a number of disciplines, including sociology, political science, and psychology. It has been studied extensively over the past three decades in a variety of cultural and political contexts, where it has been used, among other things, to explain the functioning of society and to predict collective behavior [1,2,3,4,5,6]. Moreover, research on the mechanisms and contexts of system justification has become increasingly important in recent times, when many societies have had to adapt to a changing external environment. An appropriate response in the form of societal change can be effectively moderated by the needs, interests, and actions of affected actors, stakeholders, and populations. This is why questions of system justification are so important; indeed, Vesper et al. [7] considered system justification to be the most influential theory in contemporary social psychological research.

System justification posits that people tend to justify the existing social, economic, and political order [8]. As a social science construct, system justification is therefore based on the assumed need of individuals to maintain perceived justice and legitimacy [9,10], developing an idea of social order and the mechanisms of its maintenance. Thus, members of a given community tend to defend, rationalize, and legitimize the existing social system, including its norms, rules, and practices. Most often, they do this through prejudices, stereotypes, and ideologies [11,12]. Within this framework, established social relations are considered useful and appropriate, and individual actors therefore seek to legitimize and rationalize them. An important element of system justification theory is that all population groups are motivated to engage in system justification in a cross-sectional manner, i.e., not only those who benefit from the existing social order (for whom the defense of the given order can be assumed), but also those who are disadvantaged in some way within the given social structure. In their case, system justification may represent an ex post rationalization that mitigates negative perceptions of objective inequalities and asymmetries [13,14]. System justification theory posits that the seeds of system justification attitudes are common to all individuals, but that specific actors differ in the extent to which they accentuate this attitude [15].

Empirical evidence suggests that system justification as a psychological construct is related to political conservatism [16,17,18], particularly in terms of rejection of social change. However, system justification has also been found to be related to social dominance [19,20] or patriotism [21]. In addition, some authors have pointed out that system justification may be negatively correlated with willingness to participate in protests aimed at challenging the existing social order and structure [22,23,24]. Perceived justice and legitimacy of the system have been found to reinforce stabilizing social mechanisms and inhibit social change. Individuals who support the existing system and emphasize its legitimacy internalize social inequalities and denigrate or reject alternative worldviews [25,26]. In addition, higher levels of system justification may be associated with higher perceived quality of life [27,28,29].

Research on the causes, context, and consequences of system justification is linked to the development and refinement of relevant research methods and tools. Because system justification is often masked by stereotypes [30] and ideologies [15], it (system justification) cannot be studied directly. Because system justification is not directly reflected in a particular pattern of behavior, it cannot be observed. Therefore, research on this construct requires the use of an appropriate research instrument in the form of a specific scale that is both sufficiently sensitive, reliable, and valid. Given the crucial role of perceptions of social order and its evaluation, the System Justification Scale (hereafter referred to as the SJS) was developed by Jost and Banaji [8] as a useful tool for measuring issues related to inequalities and the legitimacy of the status quo.

The SJS is a self-report research tool designed to assess the level of perceived justice, fairness, and importance of a stable social order. The developed scale can be used to determine perceptions of country conditions, assessments of the need for social change, and perceived congruence between the actual situation and individual norms or values [8]. The proposed scale recognizes that the level of system justification may vary depending on specific situational factors [31,32]. Therefore, a careful analysis of system justification in different social and cultural contexts is necessary.

Although the SJS has been used in many countries around the world during its 30 years of existence, to the best of our knowledge, it has not been validated in the context of Czech society. Therefore, the aim of this study was to fill the existing knowledge gap and to verify the applicability of the scale in the Czech society. The accomplishment of this research task involved a number of activities, including translation of the SJS into the Czech language, primary data collection, and special analyses focused primarily on the psychometric properties of the scale. In addition, the study used some novel indicators for examining construct validity that, to our knowledge, have not been used before (compliance with legal norms, acceptance of the rule of law, or civic engagement). However, the research objective reflects the specific focus of this study, which directs its main attention to the measurement instrument itself. Thus, it is primarily concerned with examining the appropriateness of the individual items of the scale, evaluating the structure of the scale, its unidimensionality, and, last but not least, a thorough analysis of the psychometric properties of the scale. These objectives are expressed in instrumental form by the specific hypotheses that this study seeks to answer. The following hypotheses were tested:

(a) System justification, as examined by the SJS, is a unidimensional construct.
(b) All SJS items are highly correlated with each other.
(c) The psychometric properties of the SJS indicate a good fit of the tested construct to the empirical data.

## 2. Materials and Methods

### 2.1. Participants and Procedures

Given the aim of the research, it was desirable to obtain findings on the SJS from a representative sample of the country’s general population aged 18–79 years. For this reason, an address-based sampling technique was used to select houses and apartments in predefined primary territorial units. The corresponding list of houses and dwellings was compiled during the census, so these data represent the best available sampling frame in terms of accuracy and completeness. In each household, specific respondents were identified using the Kish table [33]. In this way, a total of 2826 respondents were asked to participate during November 2021, and a total of 1438 face-to-face interviews were conducted; the response rate was thus 50.9%. Given that some interviews were incomplete, the final sample used in the analysis includes 1419 cases, which can be considered sufficient for the purposes of scale validation. The specific structure of the sample in terms of gender, age and size of place of residence is presented in Table 1.

### 2.2. Research Ethics

Informed consent was always obtained from participants prior to each interview. To ensure confidentiality of answers, all responses were anonymized and are presented only in summary form to avoid direct or indirect identification of specific individuals. In the case of participants aged 18–21, informed consent was obtained not only from the respondents interviewed, but also from at least one parent or guardian who was present throughout the interview.

### 2.3. Translation of the Instrument

An important step in the development of the research instrument was the translation of the SJS into the Czech language. This process was carried out carefully due to its critical importance, following the recommendations of Sousa and Rojjanasrirat [34] and Yu, Lee, and Woo [35]. Thus, two simultaneous translations were performed, with the original English version of the SJS being translated into Czech by two independent translators. The two translations were then compared and the differences identified were subsequently resolved resulting in a consolidated form of translation of the scale. A third translator then performed a back-translation from Czech to English to confirm the equivalence of the two language versions. In addition to the wording of the individual statements, the wording of the alternatives, i.e., the way in which the degree of agreement with each item was expressed, was also taken from the original scale.

The translated scale was then pilot-tested on a sample of 23 respondents recruited from the target population. Cognitive interviews in the form of think-alouds [36] were used to test the clarity of the items and their difficulty from the respondents’ perspective. The aim of these cognitive interviews was to identify any problematic, misleading or otherwise inappropriate wording. Based on the interviews conducted, one item that was perceived by respondents to be a double-barreled question was modified. Specifically, the item “Everyone has a fair chance at wealth and happiness.” Was modified to retain only “wealth” while omitting “happiness”. The reason for this was that respondents did not perceive “wealth” and “happiness” to be related, but rather pointed out their differences. In addition, only minor wording changes were made in the English translations of three other items. No other methodological problems or significant deficiencies in the clarity or accuracy of the scale items were found. The research instrument was subsequently supplemented with additional socio-demographic and merit questions.

### 2.4. Measures

In addition to the SJS, five independent variables that are substantially related to the construct of system justification were used to test the construct validity of the scale. They are explained in more detail along with the introduction of the SJS itself.

#### 2.4.1. System Justification Scale (SJS)

As mentioned above, Jost and Banaji [8] developed the SJS to measure respondents’ attitudes and perceptions regarding social order. The SJS consists of a total of eight items relating to, among other things, perceived fairness, assessment of the functioning of the existing social system, quality of life, and opportunities to access wealth. The individual items were rated on a four-point Likert-type scale ranging from 1 = strongly disagree to 4 = strongly agree, in accordance with the findings and recommendations of Roccato et al. [37]. Thus, the total score on the scale ranges from 8 to 32, with higher scores indicating higher levels of system justification. The developed scale has been repeatedly used in many studies (e.g., [1,2,3,37,38]) to assess its reliability and validity.

#### 2.4.2. Control Indicators

Given that system justification is intended to reflect the legitimacy of the existing social system and the acceptance of the status quo, even though it may not be advantageous for a given individual at a given time [15], the level of agreement with the statement “Even bad laws should be obeyed.” was measured. The initial assumption was that the degree of agreement with a given statement would be positively correlated with system justification. Next, perceived fairness was examined by measuring agreement with the statement “Ordinary people do not get a fair share of the national wealth.” In this case, a negative association was hypothesized if people with higher SJS scores were expected to agree with the statement to a lesser extent than people with lower SJS scores. The legitimacy of social order (or a rule-of-law principle) was also tested by the statement “There are different rules for rich people and different rules for poor people.” Again, a negative association was expected, with people with higher SJS scores less likely to agree with the statement than people with lower SJS scores. An important perspective that might have differentiated SJS well is the willingness to strike. Vesper et al. [7] consider this indicator to be a key element in examining the construct validity of the SJS. Given that the indicators presented above could lead to the misleading conclusion that system justifiers, i.e., individuals with high levels of system justification, are rather passive, the statement: “It is important for me to participate in preserving the environment in which I live.” was also included in the control indicators. All of these control indicators are presented in Table 2.

### 2.5. Data Analysis

All statistical analyses were performed with IBM SPSS ver. 27 except for confirmatory factor analysis, for which AMOS 24 was used.

In order to provide comprehensive information about the sample and the responde;nts involved in the research, a series of descriptive statistics were calculated: for each item of the scale, the distribution of responses was examined and the results obtained were summarized using basic statistics in the form of mean (M), standard deviation (SD), skewness, and kurtosis. The internal consistency of the scale was assessed using Cronbach’s alpha [39,40,41].

To assess the construct validity of the translated SJS, appropriate methodological recommendations and procedures were consistently followed [42]. Exploratory factor analysis (EFA) based on principal component analysis [43] was used to assess the dimensionality of the scale. However, EFA provided important evidence not only in terms of the number of dimensions (corresponding to the number of factors) and the total amount of variance explained, but also in terms of the construct validity of the scale. Therefore, factor loadings (FL) and communality data (h^2^) were used to assess concurrent validity. Factor loadings, which indicate the degree to which a variable was associated with a factor, indicated the strength of the relationship between the factor and the variable. For convergent validity, factor loadings of individual scale items should be high. In addition, communalities, which document the extent to which the total variability of a variable is explained by the factors in the analysis, indicate how the variable is explained by the identified factors. Therefore, a higher communality value indicates a higher convergent validity. Confirmatory factor analysis (CFA) was then performed using maximum likelihood estimation methods. In order to obtain a more accurate assessment of the model’s fit to the data, a number of indices were generated, namely the root mean square error of approximation (RMSEA), as well as the standardized root mean square residual (SRMR), the comparative fit index (CFI), the Tucker–Lewis index (TLI), and the GFI. In the context of conducting exploratory and confirmatory factor analysis, it should be noted that the total sample was randomly divided into two equivalent halves using a randomization procedure, as recommended by other researchers (e.g., [44]), which ensured the equivalence of the two subsamples. Subsequently, an exploratory factor analysis was conducted on the first subsample (*n* = 709), while the second subsample (*n* = 710) was used for confirmatory factor analysis. The above procedure has been successfully applied in other scale validation studies (e.g., [45]). In conducting the factor analysis, missing values were handled using the listwise method; it should be emphasized that the analysis of other variables (particularly the control indicators) may be based on a different number of valid cases.

Due to the nature of the data, tests of correlations between scale items were performed using Pearson’s correlation coefficient. An analysis of variance (ANOVA) was performed to determine if there were statistically significant differences between the groups based on the SJS scale and the control indicators, or Kendall’s tau_b values were calculated with respect to the types of variables given.

## 3. Results

### 3.1. Univariate Statistics and Internal Consistency

Table 3 provides information on the mean scores of the individual items that make up the SJS; the table also includes standard deviations. The mean scores range from 2.22 to 2.78, and the standard deviations range from 0.775 to 0.851, indicating that no score is significantly different from the others. The floor effect was 1.8% and the ceiling effect was 0.0%; the values obtained do not exceed the threshold recommended by Cain et al. [46], which is 50%. The overall mean SJS was 18.13 and the standard deviation was 4.815.

**Figure 1:**
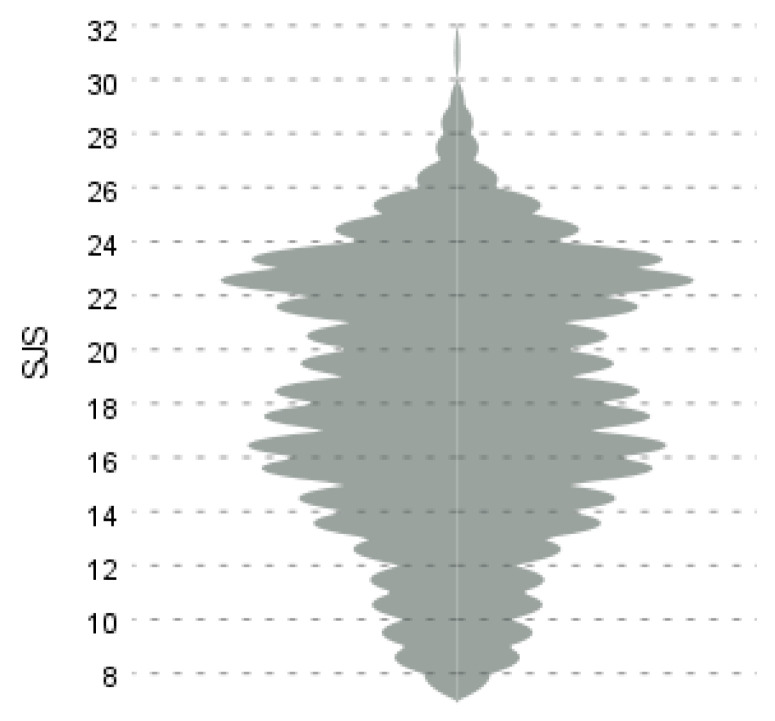
System justification scale distribution.

As further shown in Table 3, the SJS in this study reached a skewness of −0.142 and a kurtosis of −0.740; both values fall within the recommended range of −1.5 to +1.5 [47], and thus the scale can be considered normally distributed. The actual distribution of the scale scores is shown in Figure 1.

The unidimensionality of the scale was assessed using the results of exploratory factor analysis. The value of the Kaiser–Meyer–Olkin (KMO) test of sampling adequacy was 0.901, which can be interpreted as showing that the analyzed data were suitable for exploratory factor analysis. In addition, Bartlett’s test of sphericity revealed a statistically significant result with χ^2^ = 2570.627 (df = 28, *p* < 0.001). A factor with an eigenvalue >1 was extracted using principal component analysis, which explained 54.5% of the total variance. Table 3, with the factor loadings and communalities, shows that the absolute values of the factor scores ranged from 0.601 to 0.875. According to Pett et al. [48], higher absolute values indicate a greater contribution of the item to the explanation of the factor, while factor loadings greater than 0.8 indicate that the item contributes significantly to the explanation of the factor. Thus, based on the evidence presented, it can be summarized that the items comprising the SJS were interrelated through a common factor [49] and the results obtained support the hypothesis of the unidimensionality of the SJS.

The above statement is further supported by the results of the internal consistency assessment of the SJS. In this regard, Cronbach’s alpha coefficient was calculated and reached a high value of 0.884, which indicated the high internal consistency of the tested scale. This is further evidenced by the high item–total correlation (ITC), where, according to Taherdoost [50], a value of the coefficient higher than 0.4 can be considered high enough. Table 3 shows that in this study, the correlations between the items and the total scale value ranged from 0.553 to 0.815, which exceeds the recommended threshold and thus supports the conclusion that the tested scale is internally consistent. Thus, the above results provided credible and sufficient evidence that the scale items reflect the intended theoretical construct with a high degree of probability.

### 3.2. Psychometric Properties of the SJS

**Figure 2:**
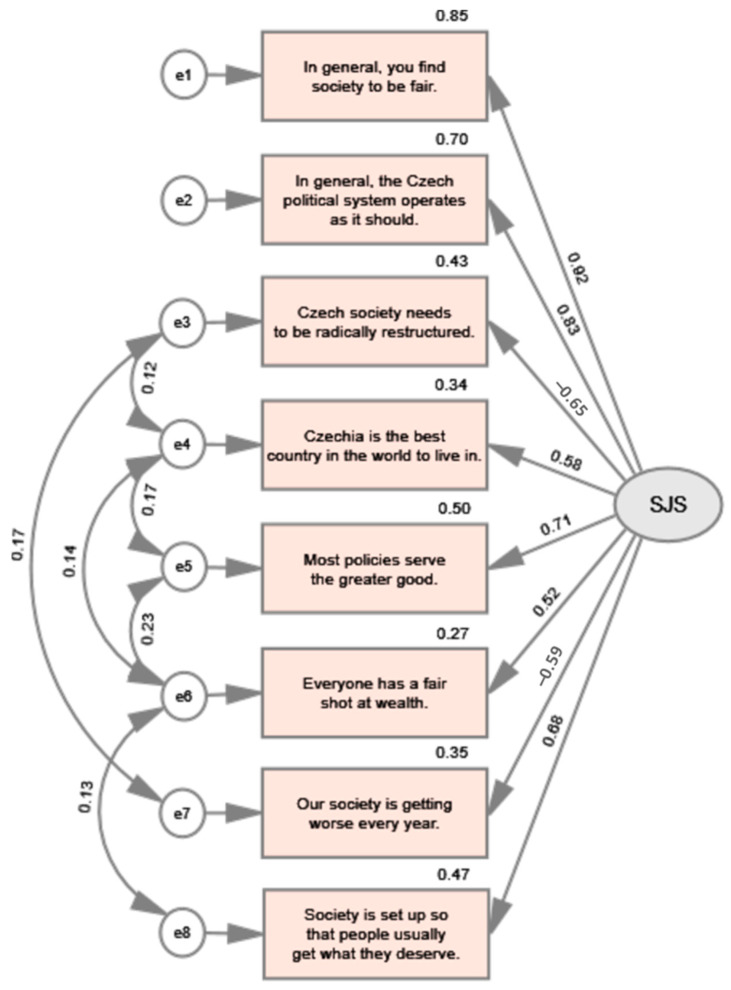
Confirmatory factor analysis (SJS) of the improved model.

The construct validity of the scale and its psychometric properties were assessed using confirmatory factor analysis (CFA) with maximum likelihood estimation. A separate subsample independent of the data used for exploratory factor analysis was used for this analysis. The results for this separate subsample (*n* = 710) showed a significant chi-square value of 30.227 (df = 14, *p* < 0.05), which is common for large samples of several hundred cases [51]. A graphical representation of the corresponding SJS model is shown in Figure 2 that introduces the standardized coefficients (factor loadings) ranging from −0.65 for the item “Czech society needs to be radically restructured” to 0.92 for the item “In general, you find society to be fair”. Moreover, the communalities are presented as well.

As the analysis progressed, the original model was adjusted to account for errors representing unobserved variables that affect variance not captured by the latent construct. Table 4 shows the results of both the original and modified models, as well as the specific absolute and incremental indices that were calculated. The results indicate a good fit and show that the proposed model fits the empirical data well.

It can be seen that the main goodness-of-fit indices provided convincing support for a good fit: the root mean square error of approximation (RMSEA) reached 0.041, which is below the recommended threshold. The standardized root mean square residuals (SRMR) also reached an excellent value of 0.0206. In addition, the goodness-of-fit index (GFI), comparative fit index (CFI), Tucker–Lewis index (TLI), and normalized fit index (NFI) also provided strong enough arguments to confirm the hypothesis of excellent fit. Their values are shown in Table 4.

### 3.3. Construct Convergent and Divergent Validity

Construct validity is crucial in the process of validating a research instrument as it informs how accurately a given scale measures the intended construct [52]. Construct validity examines the extent to which scale items are correlated with each other, as a high correlation between them indicates that they measure the same construct in a consistent manner [53]. A combination of the following tests and analyses was used to test the construct convergent validity of the Czech version of the SJS:

(a) Exploratory factor analysis; in addition to the previously mentioned results regarding factor loadings (FL) and communalities (h^2^) in Table 3, low or no cross-validation in the EFA indicated high construct convergent validity. The latter is high because there is only one factor.
(b) The average variance extracted (AVE), which represented the average amount of variance captured by the indicators associated with the latent variable, reached 0.54. The value obtained indicated that, on average, 54% of the variance of the items was explained by the latent variable; the result also indicated a high (acceptable) convergent validity given that the items were interrelated and measured the same underlying construct.
(c) Composite reliability (CR), which tested for correlations between indicators of the latent variable. The CR value in this case was 0.76, which exceeded the recommended value of 0.7. Thus, this result also indicated that the items were correlated with each other, which supported the hypothesis of the satisfactory convergent validity of the SJS and suggested that the scale was a reliable instrument in this case.
(d) The correlation matrix presented in Table 5 shows statistically significant associations between all items, suggesting that all items may be measuring the same construct. Thus, the correlation matrix provides another strong argument to support the construct validity assumption of the scale.

(e) Table 6 shows that respondents who agreed with the statement about the need to comply with the laws, even if they are bad, had higher scores on the SJS. Furthermore, the expected negative association between the SJS and perceived fairness is evident in the empirical data, with respondents who agreed with the statement that ordinary people do not get a fair share of the national wealth scoring lower on the SJS. Similarly, those who agreed with the statement that different rules apply to rich and poor people had lower SJS scores. Consistent with the expectation embedded in social justification theory, the significantly lower willingness to strike of people with high SJS scores is evident, confirming their resistance to activities aimed at delegitimizing the existing order.

An indicator of divergent validity is the willingness of people with high SJS scores to participate in activities aimed at preserving their environment. The obtained results are consistent with the hypotheses and allow us to conclude that the SJS in the Czech environment measures the intended theoretical construct.

## 4. Discussion

The main aim of this study was to evaluate the psychometric properties of the Czech version of the SJS. To this end, a combination of several analytical procedures was used, including an assessment of the scale’s internal consistency, exploratory factor analysis, and confirmatory factor analysis. Overall, the Czech version of the SJS was found to have excellent psychometric properties and to be a reliable and valid instrument for measuring system justification. Specifically, the results indicated that the SJS performed very well in the Czech context: the scale showed acceptable skewness and kurtosis [47] and the inter-item correlation values exceeded the recommended threshold of 0.4, which met the criteria for acceptability [49]. The SJS also demonstrated a unidimensional structure, high internal consistency, and satisfactory construct validity. The standardized root mean square residuals (SRMR) reached 0.0206, which was within the acceptable range [54]; the root mean square error of approximation (RMSEA) reached 0.041, which also exceeded the recommended threshold of 0.080 [55]; and the comparable fit index (CFI) and the Tucker–Lewis index (TLI) also exceeded the recommended threshold of 0.95, indicating a high level of model fit to the empirical data [54].

In addition to the exploratory factor analysis results and the value of the correlations, convergent validity was also demonstrated by the average variance extracted (AVE) and composite reliability (CR), which exceeded the recommended thresholds of 0.5 and 0.7, respectively [56,57]. In addition, the scale showed the expected (and justified) associations with each of the control indicators included in the study. The results showed, among other things, that there is a statistically significant association between the SJS and agreement with the statement that even bad laws should be complied with. This statement is one of the key elements of the convergent validity of the SJS because it is consistent with the theoretical framework, which posits that people tend to defend and justify the existing order, even when laws or structures appear imperfect or unjust.

The data also showed that people with higher SJS scores downplayed inequalities in the distribution of wealth. Again, such an interpretation is consistent with system justification theory, in that people who support and justify the current system (i.e., those with higher SJS scores) crowd out issues of inequality and fairness in distribution of wealth. Thus, the statement supports the convergent validity of the SJS scale by finding a statistically significant association between responses to this statement and the level of system justification as measured by the SJS scale.

The ANOVA results showed a statistically significant difference between the different levels of agreement with the statement about different rules for rich and poor people and the SJS score. Such a result is consistent with system justification theory, which posits that people tend to justify and legitimize the existing social order, even though it may contain inequalities and differential access to rights. Thus, the third statement documented the validity of the SJS scale by empirically confirming the expectations based on system justification theory.

Furthermore, the results showed that people’s willingness to strike was related to different levels of system justification. As expected, higher SJS scores were related to lower willingness to strike. Indeed, willingness to strike implies disagreement with some aspects of the current system or working conditions, and so it is not surprising that people who justify the current system are more likely to resist activities that inherently challenge or criticize some aspect of the current system.

The lower willingness to strike, however, cannot be equated with passivity and disinterest among people with high SJS scores. On the contrary, ANOVA results show that there is a statistically significant difference between SJS scores and commitment to engage in activities aimed at preserving the current environment. It appears that respondents with higher SJS scores consider personal involvement and efforts to preserve the environment as part of a functioning system. The results resemble the statement that people tend to justify and legitimize a system that ensures its stability by encouraging citizen participation and supporting their efforts to participate.

Given the cross-sectional nature of the research, it is important to note that it was not possible to determine the direction of association between system justification and other attitudinal indicators. For example, it is not clear whether higher levels of compliance led to higher levels of system justification or, conversely, whether higher levels of system justification led to higher levels of compliance. Clearly, both of these explanations have merit, but a different research design would be needed to clearly establish causality.

Another limitation of the research conducted stems from the fact that only a limited number of control variables were observed in the present research. It would be useful to use a wide range of behavioral questions to better describe the conditions and circumstances from which individuals form their attitudes toward the social system.

Despite these limitations, the findings may have a number of practical implications that can contribute to a better understanding of human behavior, social phenomena and processes. Understanding system justification can help to better assess people’s responses to political and social change. Knowledge of the extent to which people justify or reject the current system can be useful in designing social programs, initiatives, and campaigns aimed at social change. Knowledge of system justification can help to better understand some of the cognitive biases that may influence the way people perceive information, interpret events, and formulate opinions. Moreover, system justification can provide a useful perspective on the mechanisms that contribute to the reproduction of social inequalities. Finally, understanding the mechanisms of system justification can help identify ways to raise awareness of social problems and motivate advocacy for social justice.

## 5. Conclusions

Understanding attitudes related to system justification is crucial for effective responses to contemporary social and economic change. The results provide interesting and actionable insights into people’s perceptions of the current social, economic, and political order, the extent to which they tend to question fundamental social values, or the extent to which they support the status quo.

The SJS is an easy-to-use research instrument that provides valuable information about system justification; based on the research conducted and the empirically validated properties of the scale, the SJS can be recommended for use in future research. The SJS has demonstrated very good psychometric properties in the Czech environment. The establishment of baseline values for the scale and its individual items can serve as a starting point for many other comparisons. In the future, it would be advisable to conduct separate research that would examine in detail the associations of system justification with specific behavioral patterns and conduct robust concurrent validation; an experiment to establish criterion validity or a comparative analysis of different populations would also be appropriate. Specific research attention could also be focused on a meta-analysis or systematic-review study comparing system justification according to SJS scores in countries where the scale has been used.

The findings presented above are useful not only for political science researchers or public policy makers, but also for other stakeholders, as they add to the body of knowledge on system justification and provide specific insights into the attitudes of the population.

## Figures and Tables

**Table 1:** Socio-demographic details of respondents.

Variable		Theoretical Population *	Sample (%)
Gender	Male	50.0%	50.2%
	Female	50.0%	49.8%
	Total	100.0%	100.0%
Age	18–29 years	17.1%	16.9%
	30–39 years	17.6%	17.5%
	40–49 years	21.2%	21.4%
	50–59 years	16.2%	16.3%
	60–79 years	27.9%	27.9%
	Total	100.0%	100.0%
Size of place of residence	Less than 10,000 inhabitants	46.1%	46.4%
	10,000 to 19,999 inhabitants	9.0%	9.1%
	20,000 to 49,999 inhabitants	13.0%	12.8%
	50,000 to 99,999 inhabitants	9.0%	9.0%
	100,000 inhabitants or more	22.9%	22.7%
	Total	100.0%	100.0%

* Data about the theoretical population come from the Czech statistical office.

**Table 2:** Distribution of the responses to control indicators.

Variable		Definitely Agree	Agree	Disagree	Definitely Disagree	*n*	Mean	SD
1	Even the bad laws must be complied with.	21.0%	38.9%	26.3%	13.8%	692	2.67	0.959
2	Ordinary people do not get a fair share of the national wealth.	25.0%	47.3%	23.0%	4.7%	677	2.92	0.815
3	There are different rules for rich and poor people.	33.9%	41.2%	20.5%	4.4%	687	3.05	0.847
4	I would strike for better living conditions.	12.7%	36.3%	42.0%	9.0%	691	2.47	0.827
5	It is important for me to participate in preserving the environment in which I live.	21.6%	43.8%	26.7%	7.9%	700	2.79	0.869

Note: *n* differs due to uneven number of missing cases.

**Table 3:** Summary statistics and item analysis of the system justification scale (SJS).

		Mean	SD	Skewness	Kurtosis	ITC	FL	h^2^
1	In general, you find society to be fair.	2.30	0.795	−0.059	−0.644	0.815	0.875	0.77
2	In general, the Czech political system operates as it should.	2.34	0.785	0.039	−0.470	0.747	0.830	0.69
3	Czech society needs to be radically restructured. (R)	2.74	0.848	−0.120	−0.677	0.597	0.795	0.63
4	Czechia is the best country in the world to live in.	2.25	0.851	0.210	−0.596	0.574	0.758	0.57
5	Most policies serve the greater good.	2.23	0.802	0.067	−0.637	0.714	0.677	0.46
6	Everyone has a fair chance at wealth.	2.22	0.775	0.006	−0.641	0.574	0.674	0.45
7	Our society is getting worse every year. (R)	2.78	0.818	−0.208	−0.502	0.553	−0.646	0.42
8	Society is set up so that people usually get what they deserve.	2.27	0.800	0.052	−0.574	0.668	−0.601	0.43
The whole SJS scale		18.13	4.815	−0.142	−0.740			

(R) = reverse coding; *n* = 705.

**Table 4:** Absolute and incremental indices (SJS).

Indices	Critical Values	Original Model	Improved Model
RMSEA (Root Mean Square Error of Approximation)	<0.080	0.089	0.041
SRMR (Standardized Root Mean Square Residual)	<1.0000	0.0434	0.0206
GFI (Goodness-of-Fit Index)	>0.9	0.949	0.989
CFI (Comparative Fit Index)	>0.9	0.958	0.994
TLI (Tucker–Lewis Index)	>0.9	0.941	0.988
NFI (Normed Fit Index)	>0.9	0.951	0.989

**Table 5:** Correlation matrix (SJS).

Item		1	2	3	4	5	6	7	8
1	In general, you find society to be fair.	1.000							
2	In general, the Czech political system operates as it should.	0.722 **	1.000						
3	Czech society needs to be radically restructured. (R)	–0.504 **	–0.455 **	1.000					
4	Czechia is the best country in the world to live in.	0.472 **	0.404 **	–0.281 **	1.000				
5	Most policies serve the greater good.	0.594 **	0.554 **	–0.414 **	0.457 **	1.000			
6	Everyone has a fair chance at wealth.	0.463 **	0.437 **	–0.229 **	0.401 **	0.472 **	1.000		
7	Our society is getting worse every year. (R)	–0.429 **	–0.376 **	0.443 **	–0.187 **	–0.357 **	–0.230 **	1.000	
8	Society is set up so that people usually get what they deserve.	0.558 **	0.559 **	–0.293 **	0.410 **	0.479 **	0.465 **	–0.303 **	1.000

Kendall’s tau_b; ** = correlation is significant at the 0.01 level (2-tailed); (R) = reverse coding.

**Table 6:** System justification scale (SJS) measured by the control indicators.

Items		*n*	%	SJS Mean Score	SJS Score SD	F	df	*p*-Value *
Even the bad laws must be complied with.	definitely agree	145	21.0%	18.73	5.120	7.463	3	0.000
	agree	269	38.9%	18.61	4.752			
	disagree	182	26.3%	18.00	4.093			
	definitely disagree	96	13.8%	16.13	5.208			
Ordinary people do not get a fair share of the national wealth.	definitely agree	169	25.0%	16.31	5.226	11.964	3	0.000
	agree	320	47.3%	18.42	4.648			
	disagree	156	23.0%	19.12	4.054			
	definitely disagree	32	4.7%	19.47	5.224			
There are different rules for rich and poor people.	definitely agree	233	33.9%	16.38	5.059	18.451	3	0.000
	agree	283	41.2%	18.69	4.436			
	disagree	141	20.5%	19.27	3.923			
	definitely disagree	30	4.4%	19.78	5.971			
I would strike for better living conditions.	definitely agree	88	12.7%	16.53	5.513	13.026	3	0.000
	agree	251	36.3%	17.83	4.558			
	disagree	290	42.0%	18.27	4.472			
	definitely disagree	62	9.0%	21.26	4.925			
It is important for me to participate in preserving the environment in which I live.	definitely agree	151	21.6%	18.97	5.303	4.849	3	0.002
	agree	307	43.8%	18.35	4.585			
	disagree	187	26.7%	17.64	4.372			
	definitely disagree	55	7.9%	16.40	5.459			

* ANOVA.

## Data Availability

The data used to support the findings of this study will be available from the corresponding author upon reasonable request.
